# Supplementary material for: Energetic Bio-Activation of Some Organic Molecules and Their Antioxidant Activity in the Pulp of the Moroccan Argan Tree «Argania spinosa L.»
Source: Molecules. 2022 May 22;27(10):3329. doi: 10.3390/molecules27103329 (PMC9144852; doi:10.3390/molecules27103329)
Supplement: Supplementary file 1 [file molecules-27-03329-s001.zip › molecules-1708532-supplementary.pdf]

Supplementary Material

# Energetic bio-activation of some organic molecules and their antioxidant activity of the Pulp of the Moroccan Argan Tree *« Argania spinosa* L »

The methodological investigation and the HPLC chromatograms.

The HPLC was used for the identification of the major organic compounds of Agania spénosa L. using seventeen phenolic compounds as standards (Table 2 in the main manuscript). Initially, 100 g of the plant powder was extracted with methanol, and then the extract was evaporated. The sample (1 mg/mL) of the evaporated crude extract was used for HPLC analysis and its compounds were quantified by comparing them with the standards.

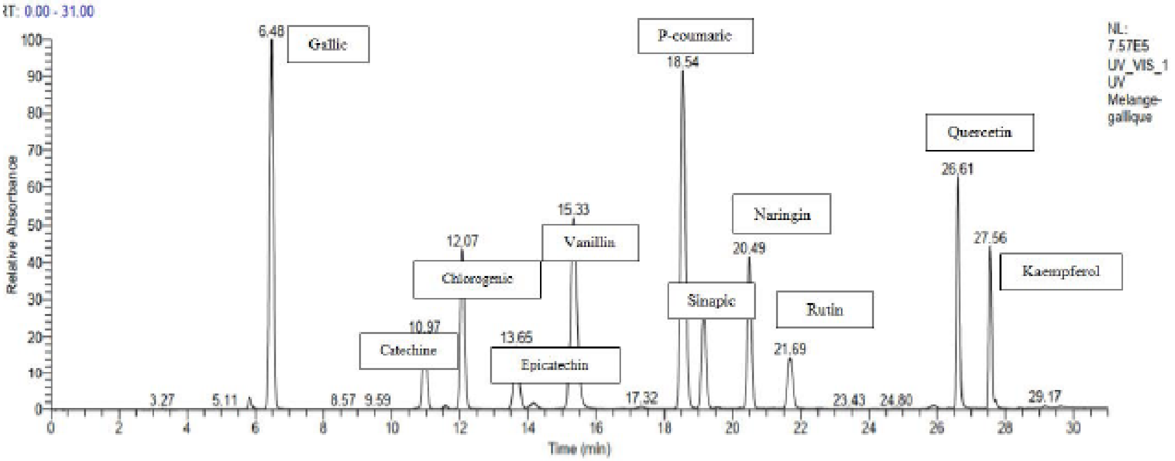


**Figure S1**. HPLC chromatogram of gall mixture standards

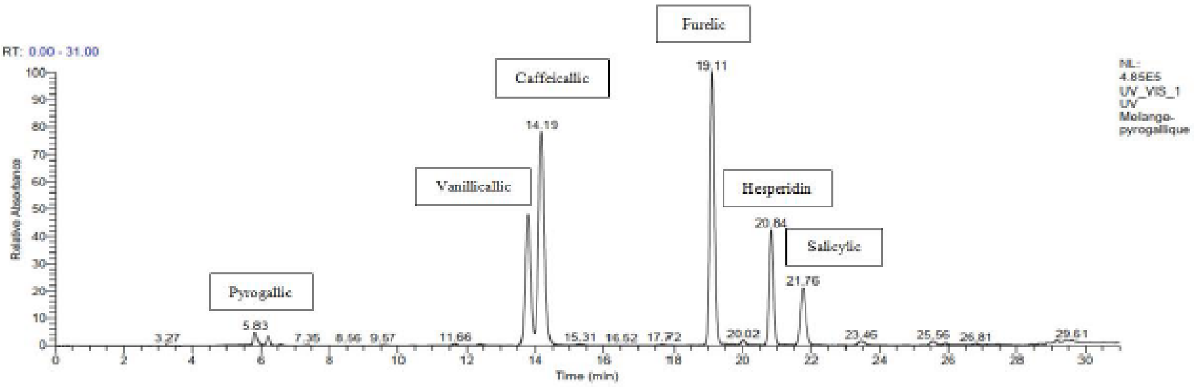


**Figure S2.** HPLC chromatogram of pyrogallic mixture standards

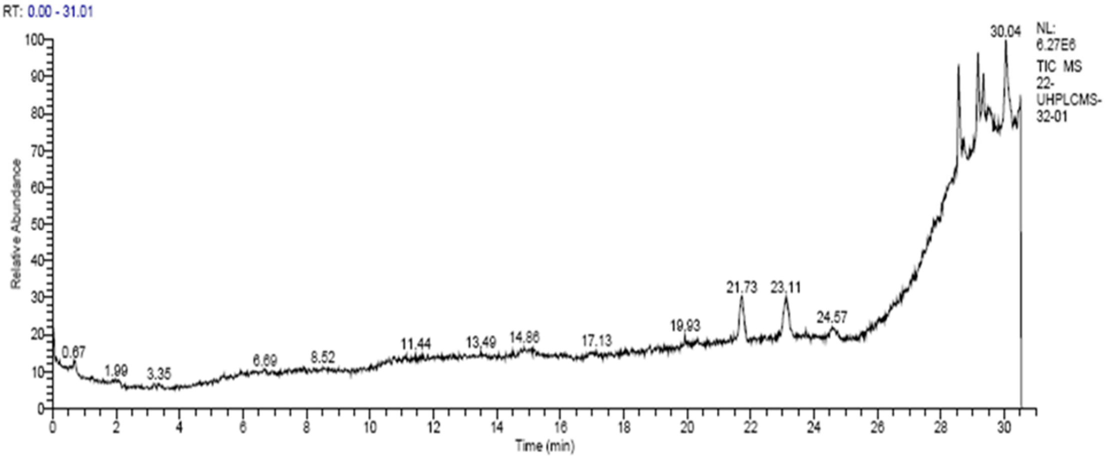


**Figure S3.** HPLC chromatogram after treatment at 25°C

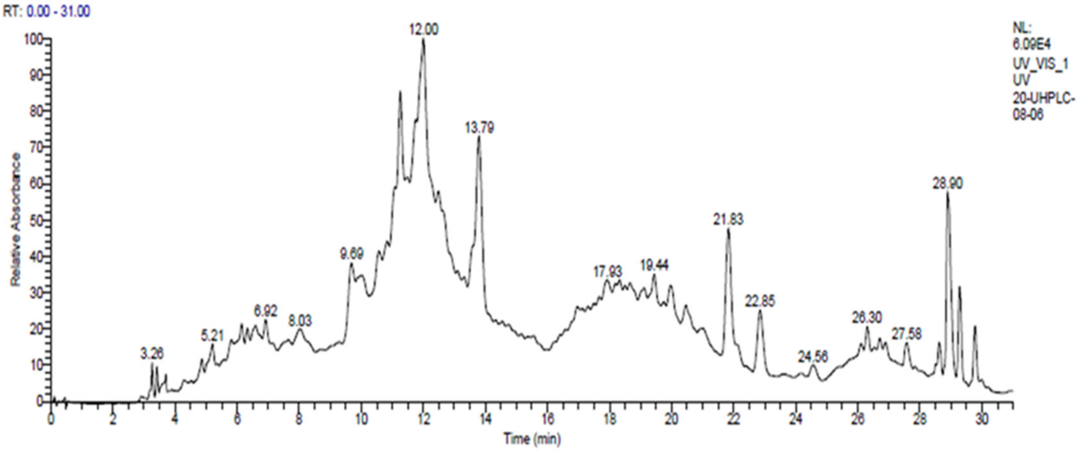


**Figure S4.** HPLC chromatogram after treatment at 40°C

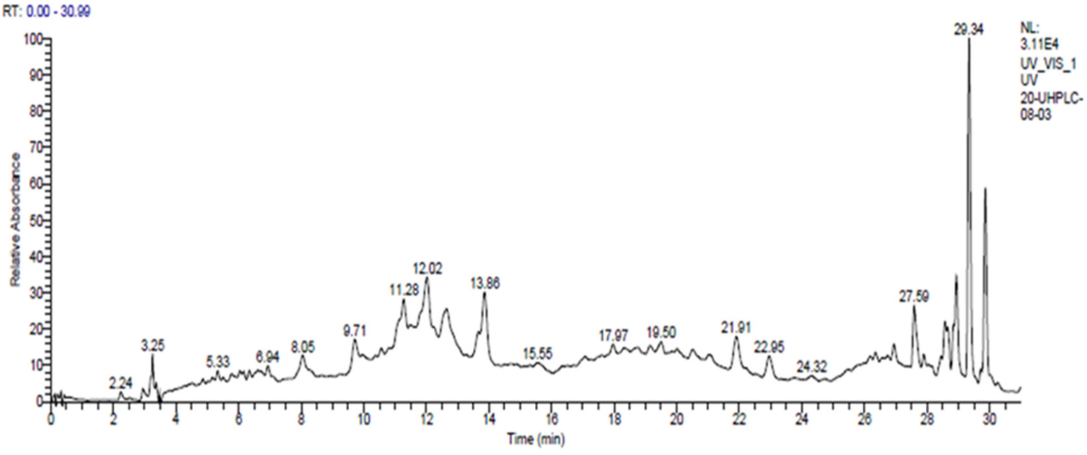


**Figure S5.** HPLC chromatogram after treatment at 50°C

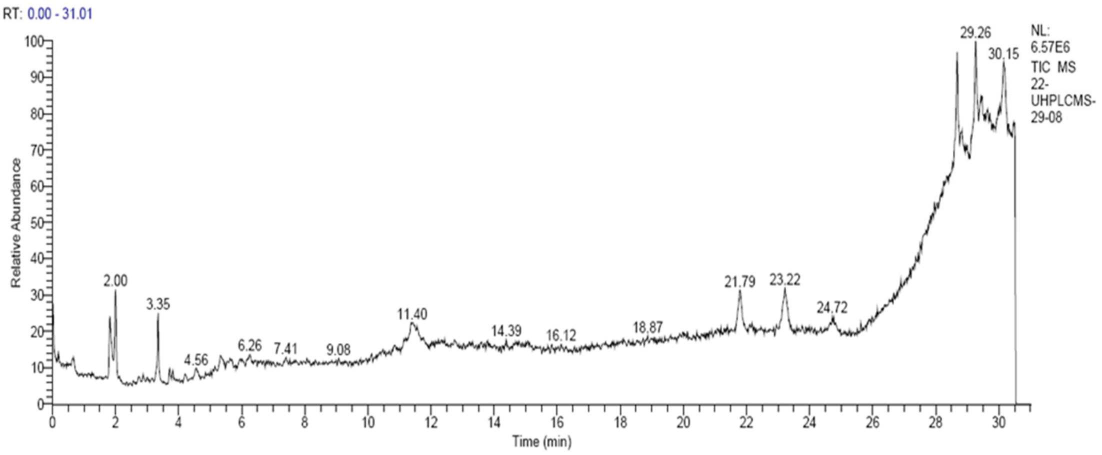


**Figure S6.** HPLC chromatogram after treatment at 60°C

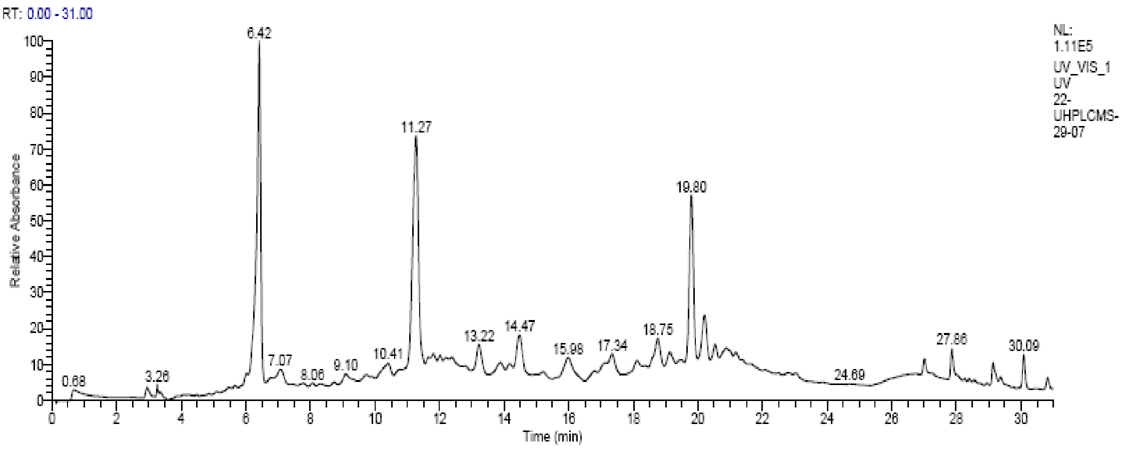


**Figure S7.** HPLC chromatogram after treatment at 70°C

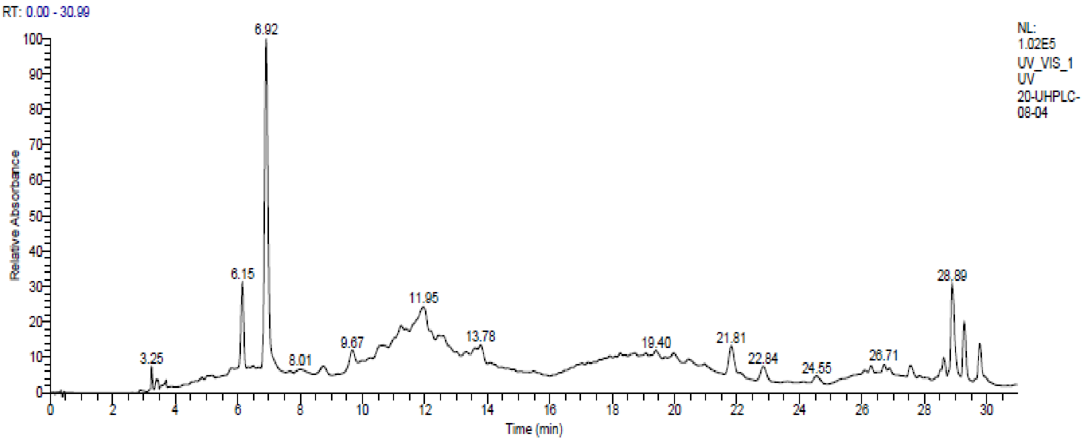


**Figure S8.** HPLC chromatogram after treatment at 80°C

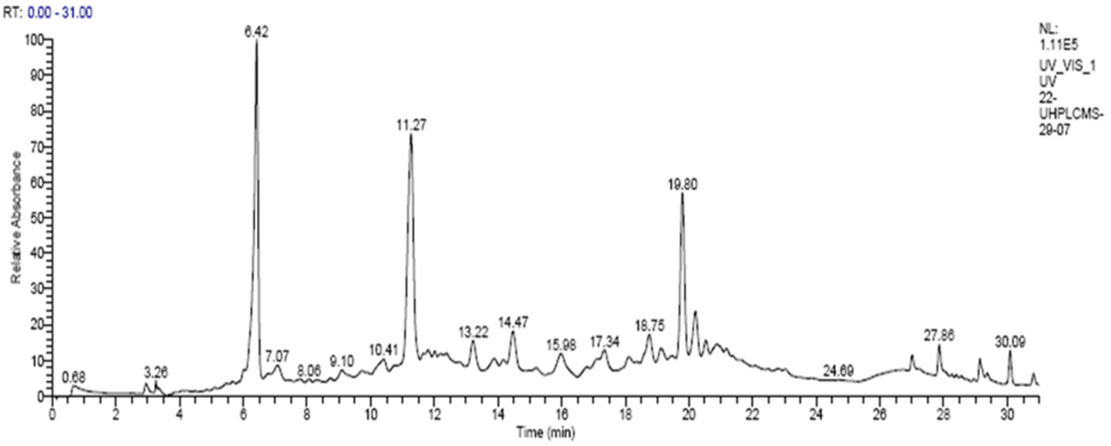


**Figure S9.** HPLC chromatogram after treatment at 90°C

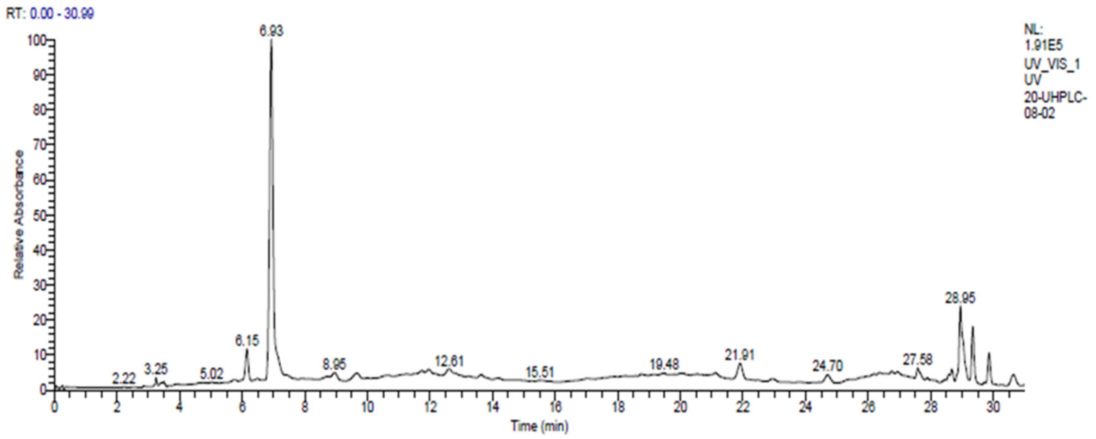


**Figure S10.** HPLC chromatogram after treatment at 100°C

**The GC-MS chromatograms at different heat temperatures**

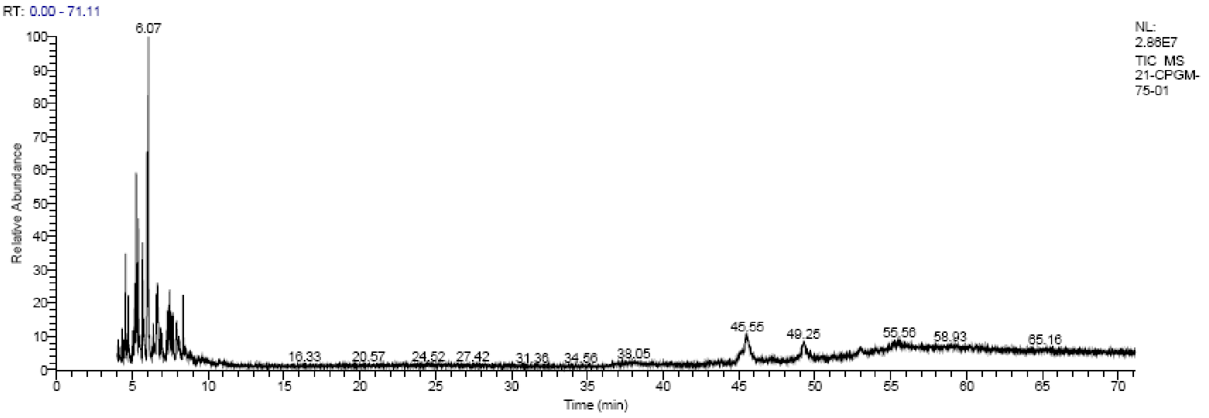


**Figure S11.** GC-MS chromatogram of heat treatment at 25°C

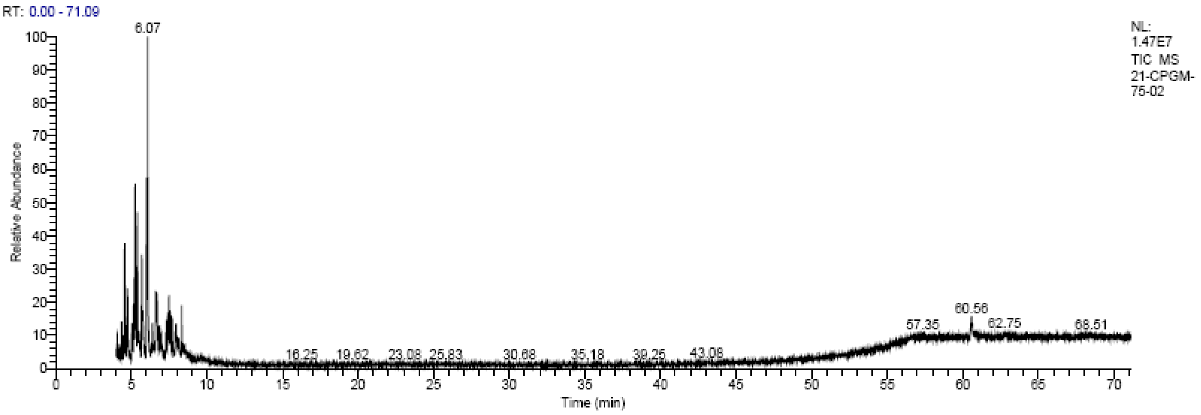


**Figure S12.** GC-MS chromatogram of heat treatment at 40 °C.

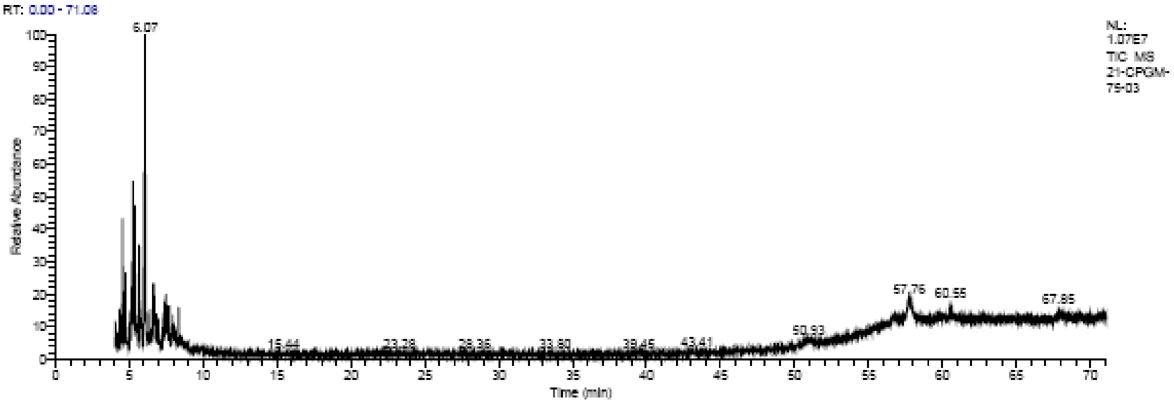


**Figure S13.** GC-MS chromatogram of heat treatment at 50°C

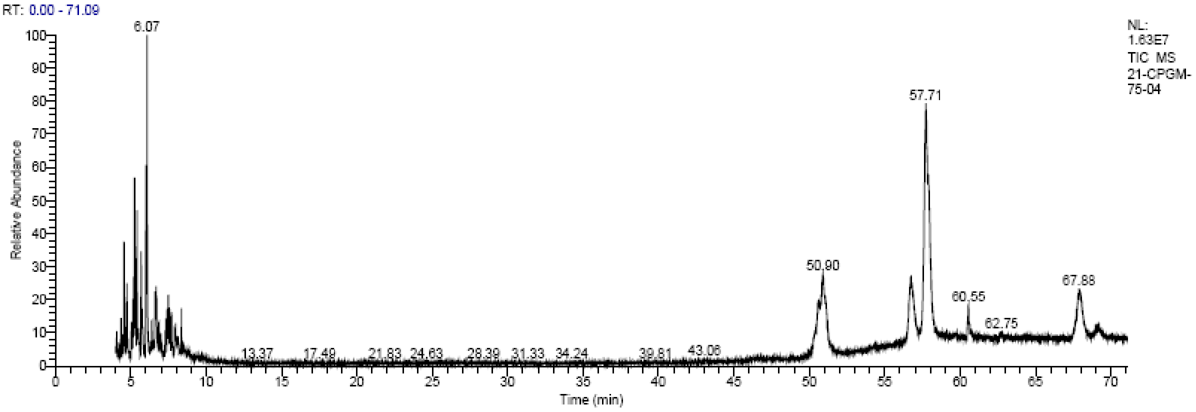


**Figure S14.** GC-MS chromatogram of heat treatment at 60°C

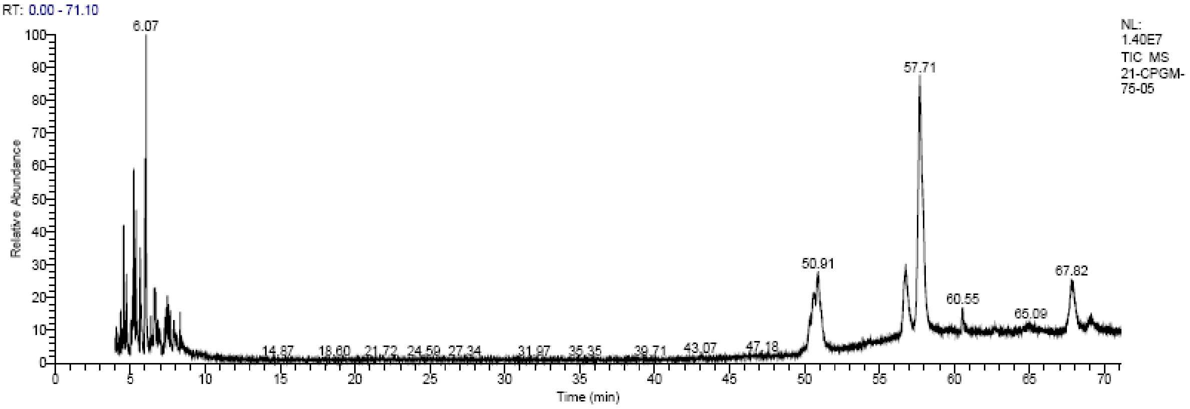


**Figure S15.** GC-MS chromatogram of heat treatment at 70°C

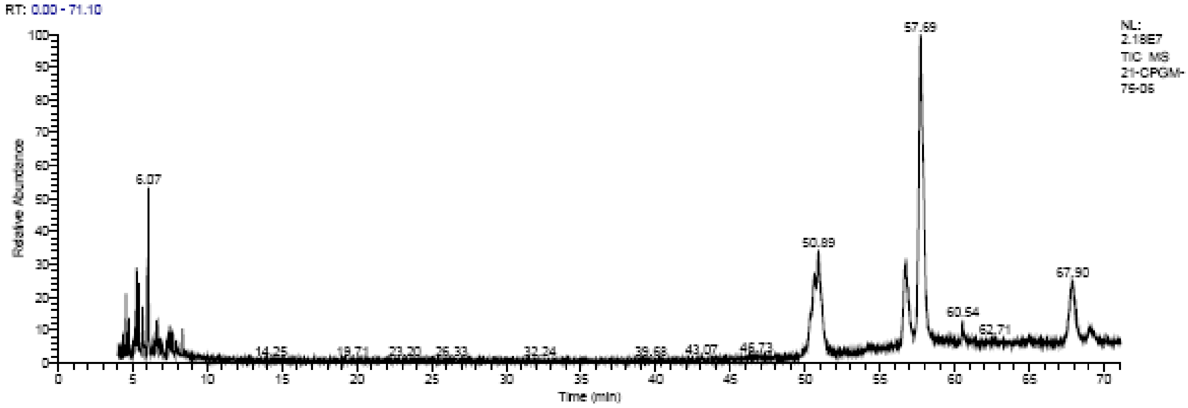


**Figure S16.** GC-MS chromatogram of heat treatment 80°C

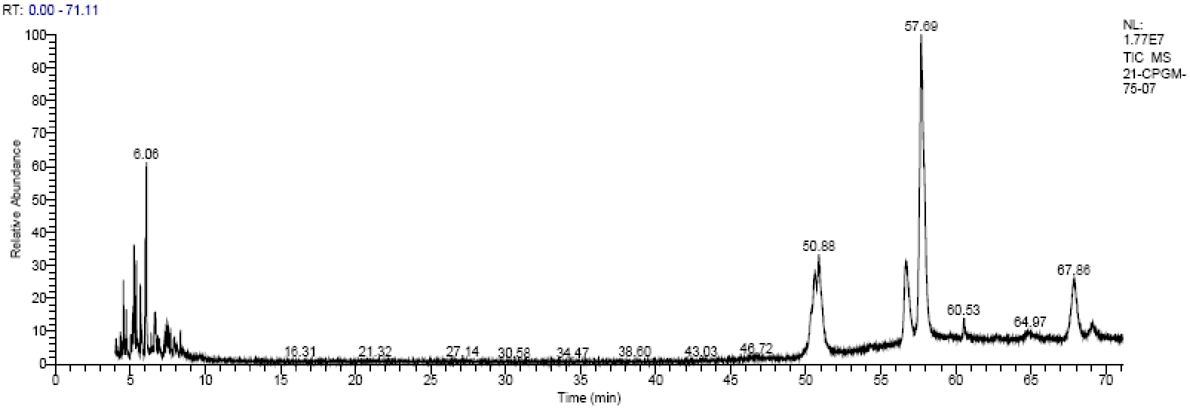


**Figure S17.** GC-MS chromatogram of heat treatment at 90°C

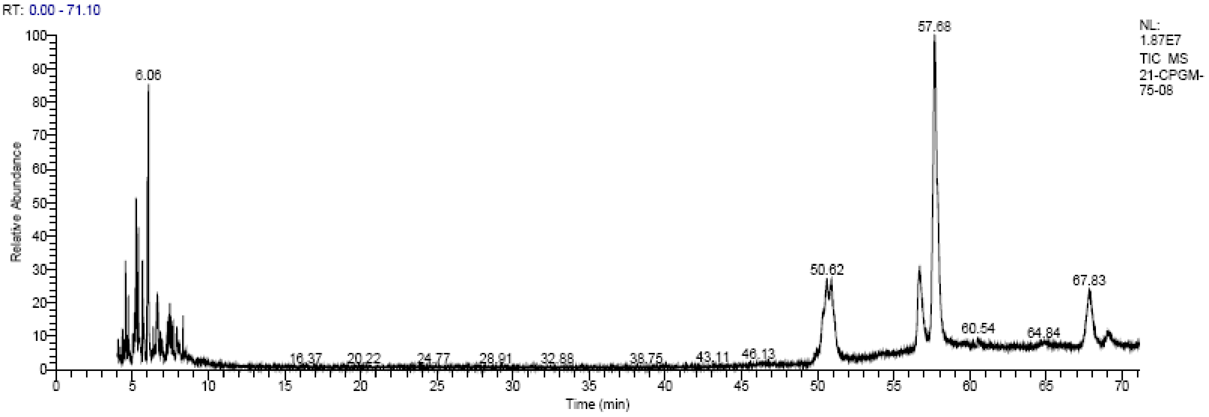


**Figure S18.** GC-MS chromatogram of heat treatment at 100°C
